# Membrane voltage as a dynamic platform for spatiotemporal signaling, physiological, and developmental regulation

**DOI:** 10.1093/plphys/kiab032

**Published:** 2021-02-03

**Authors:** Martina Klejchova, Fernanda A L Silva-Alvim, Michael R Blatt, Jonas Chaves Alvim

**Affiliations:** Laboratory of Plant Physiology and Biophysics, Bower Building, University of Glasgow, Glasgow G12 8QQ, UK

## Abstract

Membrane voltage arises from the transport of ions through ion-translocating ATPases, ion-coupled transport of solutes, and ion channels, and is an integral part of the bioenergetic “currency” of the membrane. The dynamics of membrane voltage—so-called action, systemic, and variation potentials—have also led to a recognition of their contributions to signal transduction, both within cells and across tissues. Here, we review the origins of our understanding of membrane voltage and its place as a central element in regulating transport and signal transmission. We stress the importance of understanding voltage as a common intermediate that acts both as a driving force for transport—an electrical “substrate”—and as a product of charge flux across the membrane, thereby interconnecting all charge-carrying transport across the membrane. The voltage interconnection is vital to signaling via second messengers that rely on ion flux, including cytosolic free Ca^2+^, H^+^, and the synthesis of reactive oxygen species generated by integral membrane, respiratory burst oxidases. These characteristics inform on the ways in which long-distance voltage signals and voltage oscillations give rise to unique gene expression patterns and influence physiological, developmental, and adaptive responses such as systemic acquired resistance to pathogens and to insect herbivory.

## Teaser

Membrane voltage serves as a platform coordinating ion flux to transmit and transduce biological signals.

**Advances**

- The biophysics of transport that determine membrane voltage are well-described with quantitative flux equations.
- In the models of the guard cell and the giant algae *Chara* and *Nitella* these charge-transporting processes accurately describe and predict physiological behavior, including the coupling of membrane voltage oscillations with ion flux, [Ca^2+^]_i_, pH, their consequences for cellular osmotic adjustments, and their spatial propagation.
- Unlike neuronal and other animal tissues, action potentials in plants are mediated by a temporal sequence of ion flux through Ca^2+^ and Cl^-^ channels with voltage recovery driven by ion flux through K^+^ channels. The interplay of channel-mediated ion flux and changes in H^+^-ATPase activity are likely responsible for the slower propagation of variation and systemic potentials.
- In terrestrial plants, membrane voltage transients may propagate along vascular traces, both through the parenchymal cells lining the xylem and through the phloem. Propagation of such voltage transients is associated with glutamate receptor-like channels that may contribute to plasma membrane Ca^2+^ flux and [Ca^2+^]_i_ elevations.
- Changes in [Ca^2+^]_i_, pH, and reactive oxygen species are key mediators that translate voltage signals into physiological, developmental, and adaptive responses in plant tissues.

## Introduction

Voltage is, at once, one of the simplest of membrane variables to quantify and one of the most challenging to comprehend. Its place in the collective consciousness of plant physiologists is deeply engrained, even if its origins often go unacknowledged or are misunderstood. Membrane voltage—that is the electrical potential difference across a membrane—has long been recognized in cells to arise from the transport of ions, especially by H^+^-ATPases. It contributes to the bioenergetics of the membrane, and it is a central factor regulating the activity of voltage-gated ion channels. Its dynamics have also fuelled the notion of membrane voltage as an entity in its own right that, like the cytosolic-free Ca^2+^ concentration ([Ca^2+^]_i_), signals physiological and developmental events within and between plant cells. Neither of these perceptions is incorrect per se. Indeed, membrane voltage is central to all membranes as part of their bioenergetic “currency” and it also serves important roles in cellular and long-distance signaling, some of which are only now beginning to surface. The nuancing of the detail is all-important, however; understanding the origins of membrane voltage is essential in order to grasp its significance.

Here, we address the phenomenon of membrane voltage, from its origins in the net transport of charge across the membrane to its integration in signaling events in plants. We discuss a small selection of examples to illustrate the contributions of membrane voltage to both processes and we highlight some of the questions outstanding to understanding voltage signaling in plant biology. As a short review, it is not possible to cover more than a very small fraction of current knowledge pertinent to membrane voltage in the physiology and development of plants. The interested reader may wish to consult several excellent reviews that cover transport integration, voltage-dependent gating of channels and signaling in guard cells (Lawson and Blatt, 2014; Assmann and Jegla, 2016; Jezek and Blatt, 2017), Ca^2+^ signaling (Hetherington and Brownlee, 2004; McAinsh and Pittman, 2009; Roelfsema and Hedrich, 2010), action potentials (APs) in Characean algae (Thiel et al., 1997; Shepherd et al., 2002) and the Venus flytrap (Sibaoka, 1969; Hedrich et al., 2016), and finally transport and signaling in pollen and roothairs (Campanoni and Blatt, 2007; Zonia and Munnik, 2007). We also direct the reader to a companion Update review on systemic signaling (Johns et al., 2021) and to treatments of the all-important kinetics of transport and its interpretation [see Sanders (1990) and several useful chapters in *Membrane Transport in Plants* (Blatt, 2004)].

## A historical perspective

It is well worth reviewing briefly the concepts around membrane voltage from a historical perspective. Naturalists of the 17th and early 18th centuries were absorbed by the question “From where do plants derive their biomass and what drives its accumulation?” Van Helmont (1648, translated in Howe, 1965) concluded that plants come from water alone while Woodward (1699; see Thomas, 1955) proposed that plants are derived from soil. Their studies were followed by the experiments of Stephen Hales and Joseph Priestley who identified the requirement for gas exchange to support plant growth and von Liebig (1840) who first correctly identified the need of plants for inorganic minerals, leading to considerations of their uptake. Brezeale (1906), Hasselbring (1914), and Stiles and Kidd (1919) showed that plants could regulate solute uptake independent of transpiration. Thus, by the beginning of the 20th century, selective mineral nutrition was a recognized feature of growing plants.

**Figure 1:**
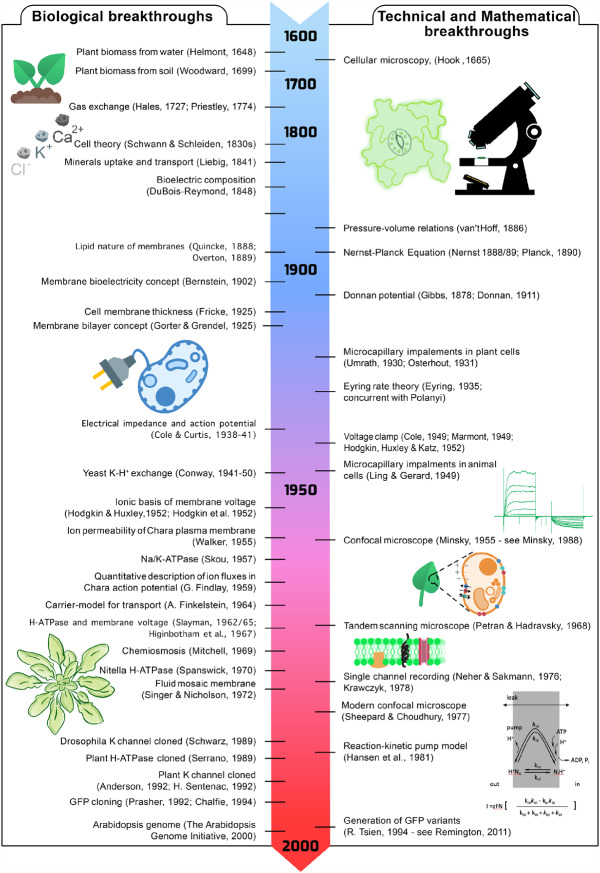
Conceptual and technical advances in understanding membrane voltage and its connections to cellular activities. A condensed timeline of discoveries, conceptual, and technical advances behind our present understanding of membrane voltage, transport, and signaling. Several of these advances are highlighted in the text.

Advances in cell biology and physics around this time (Figure 1) also marked new ideas that would come to define our thinking about cellular transport. Key among these, the eminent botanist Wilhelm Pfeffer (1877) proposed the existence of a delimiting and semi-permeable cell membrane or “skin” (Plasmahaut), a concept that derived from his studies of osmosis and nyctinastic movements of plant leaves. In the following decade, Nernst (1888, 1889) and Planck (1890) combined Fick’s law of diffusion and Ohm’s law of electrical conductance to introduce what is now known as the Nernst–Planck Electrodiffusion Equation, from which comes the Nernst Equation relating chemical and electrical driving forces across a membrane at equilibrium. Finally, Bernstein (1902) combined these concepts with the studies of his former mentor, DuBois–Reymond, and the well-known experiments of Galvani over a century earlier, to propose that the cell membrane and its ionic permeability is the basis for “bioelectricity” in cells.

Against this backdrop, the nature of membranes and membrane voltage rapidly became the focus of experimental work, notably with the large and easily manipulated cells of invertebrate eggs, the squid axon, and the giant cells of the syncitial algae *Chara* and *Nitella*. Working at Woods Hole, Cole and Curtis (1939, 1941) demonstrated that the AP of the squid giant axon was accompanied by very large changes in membrane conductance. These experiments demanded surgical removal of the axon from freshly-caught squid. Squid are seasonal along the Atlantic Coast. So, during the autumn and winter, when squid were not available, Cole and Curtis worked with the giant alga *Nitella*, noting that these cells also showed APs, albeit 1000-fold slower than those of the axon (Cole and Curtis, 1938). Cole and Curtis concluded, correctly, that the APs in both axon and alga were mediated by a large and transient rise in membrane conductance and a transmembrane flux of ions. However, they were puzzled by the fact that the voltage would overshoot zero and reverse sign by several tens of millivolts. If the membrane was permeable to all ions, however transient, then surely the voltage should simply approach zero? Clearly, the problem related to a selectivity among ions present for permeation across the membrane. The challenge was to identify which ions were permeable. Cole (1949) introduced a feedback circuit—a voltage clamp—to control membrane voltage and measure transmembrane current. However, it was Hodgkin, Huxley, and Katz (Hodgkin and Huxley, 1952; Hodgkin et al., 1952) who, in work with the voltage clamp, devised the experimental strategies that were necessary to separate and identify the current components and establish the ionic basis of the AP. Their studies showed that the membrane voltage could be explained on the basis of controlled diffusion of ions across the membrane, first of Na^+^ inward and then of K^+^ outward. The existence of these ion-selective conductances, implicit to their findings, would later come to be known as ion channels.

The idea that membrane voltage was determined by passive ionic diffusion was widely accepted by the 1960s, but was seen to relate principally to the giant cells and syncitia that were the focus of Hodgkin, Huxley, Cole, Curtis, and others. These tissues were able to survive cutting, permeabilization, and other manipulations needed to access the cytoplasmic side of the membrane. Some 40 years earlier, plant electrophysiologists had started using glass microcapillaries—microelectrodes—impaled into cells to gain access to the inside of the plasma membrane (see Umrath, 1930, 1932; Osterhout, 1931). As these new methods drew wider attention among mammalian physiologists (Ling and Gerard, 1949), animals cells were found, almost without exception, to exhibit membrane voltages close to −40 to −50 mV. When bathed in simple solutions of monovalent ions, similar values were obtained from plant cells, including the giant algae *Chara* and *Nitella* [see Hope and Walker (1975); only later would researchers understand the importance of including a small amount of Ca^2+^ in the bathing solution to reduce so-called leak conductances in plant cells]. Such voltages were largely consistent with passive diffusion of K^+^ from the cell together with minor contributions from conductances to Na^+^ and Cl^−^.

Following the Second World War, radiotracers were soon widely available to researchers. Analysis of ^22^Na^+^ and ^42^K^+^ fluxes in animals supported the idea that passive diffusion dominated the membrane voltage: the studies suggested that the gradients of these cations were maintained by a background exchange of Na^+^ and K^+^ that was all but electrically silent in animals. Even as late as the 1980s, the idea that the membrane voltage of any cell, animal or plant, might be determined by more than ionic diffusion was ridiculed in some circles. Indeed, the electrogenic nature of the mammalian Na^+^/K^+^-ATPase remained a matter of debate for decades, ending only with the studies of Gadsby and DeWeer in the mid-1980s with the demonstration of a current carried by the pump (DeWeer et al., 1988).

## Is membrane voltage a measure of pump activity?

The dogma of voltage determined by passive ionic diffusion was first challenged in 1962 by Clifford Slayman (Slayman and Slayman, 1962; Slayman, 1965) in his electrical recordings from the fungus *Neurospora*, and later by Spanswick (1970) and Higinbotham et al (1967) who extended these studies to *Nitella*, pea, and oat mesophyll cells. Slayman recorded resting membrane voltages from *Neurospora* that were typically around −200 mV, some −150 mV more negative inside relative to outside than could be explained by diffusion of any of the major ions present. Furthermore, the membrane voltage collapsed to the diffusion potential for K^+^ when respiration was inhibited with azide, dinitrophenol, or carbon monoxide. Kitasato (1968) observed the resting membrane potential of *Nitella* also was often substantially more negative than any major diffusion potential, including that of K^+^, and showed that this voltage was strongly dependent on the concentration of H^+^ in the medium. Against the backdrop of Peter Mitchell’s new ideas of chemiosmosis (Mitchell, 1969), plants and fungi were soon recognized to utilize ATPases that pumped H^+^ out of the cell, with a major proportion of the energy of ATP hydrolysis used to maintain a large transmembrane voltage, inside negative, as well as a gradient in pH. Other studies showed that the membrane voltage depolarized when uncharged nutrients such as glucose were taken up, observations that could only be explained if these solutes were transported together with an inward flux of H^+^ (Slayman and Slayman, 1974; Schwab and Komor, 1978). These findings were strong indications that transport in plant and fungal cells was coupled to the H^+^ electrochemical gradient. In retrospect, they also clearly demonstrate that membrane voltage is not simply a measure of pump activity.

Ironically, many physiological studies of the plasma membrane H^+^-ATPase and of H^+^-coupled transport also drew attention to voltage as a readout rather than to the flux of H^+^ itself, in part because of the difficulties in quantifying H^+^ flux across a coupling membrane and the buffering of H^+^ in aqueous solution. As one example of the errors arising from interpretations based on voltage alone as a readout for pump activity, we need not stray from the plasma membrane and K^+^ relations. The membrane voltage of plant and fungal cells, when K^+^ replete (and in the presence of Ca^2+^), is very sensitive to extracellular H^+^ concentration in the micromolar range (pH 5–6) and only weakly to extracellular K^+^ concentration, especially below 1 mM. However, when starved of K^+^ to reduce the intracellular K^+^ content, the same cells show membrane voltages that are strongly dependent on extracellular K^+^ concentration, even in the micromolar range, and ATP-dependent H^+^ extrusion that is enhanced in near one-to-one exchange when K^+^ is added outside. Earlier studies by Conway of K^+^-starved yeast also showed an exchange of H^+^ with K^+^ (Conway and Brady, 1950).

These, and related observations led to more than a decade of research, through the 1970s and into the 1980s, directed to uncovering a hypothetical H^+^/K^+^ exchange ATPase in plants (Leonard and Hotchkiss, 1976; Briskin and Leonard, 1982). The H^+^/K^+^-ATPase was proposed to operate in a manner analogous to the mammalian Na^+^/K^+^-ATPase and, additionally, to incorporate “slippage” that would allow for electrogenesis, rather than H^+^ exchange with K^+^, when sufficient K^+^ was present in the cell. The demonstration of an H^+^–K^+^ symport, its strong voltage dependence, and its consequent interdependence on H^+^-ATPase activity in *Neurospora* (Rodriguez-Navarro et al., 1986; Blatt et al., 1987; Blatt and Slayman, 1987) and subsequently in plants (Gibrat et al., 1990; Maathuis and Sanders, 1994) brought to a close the hunt for an H^+^/K^+^-ATPase. These high-affinity K^+^ transporters are now recognized as members of the widely expressed KT/KUP/HAK family of transporters that contribute to K^+^ nutrition and growth as well as osmotic and stress responses (Quintero and Blatt, 1997; Ahn et al., 2004; Osakabe et al., 2013; Very et al., 2014). The work with *Neurospora* also demonstrated a truly chemiosmotic coupling of high-affinity K^+^ uptake to the H^+^ electrochemical potential. The studies emphasize the importance of membrane voltage as a common intermediate shared between the H^+^-ATPase and H^+^–K^+^ symport. They also highlight the voltage sensitivity of both the symport and the H^+^-ATPase in *Neurospora* (Blatt and Slayman, 1987), a characteristic that is similarly documented for the H^+^-ATPase of *Chara* (Blatt et al., 1990) and of guard cells (Blatt, 1987; Lohse and Hedrich, 1992).

## Voltage is a shared kinetic intermediate in transport

The fundamental interdependence in activities of the H^+^–K^+^ symport with the H^+^-ATPase clearly shows how voltage connects all charge-carrying transporters within a single membrane. Quite simply, voltage acts both as a driving force for transport—an electrical “substrate”—and as a product of charge flux across the membrane. In the case of high-affinity K^+^ uptake by fungi and plants, we can think of voltage as a product of the H^+^-ATPase and a substrate for K^+^ uptake by the H^+^–K^+^ symport^*^. Furthermore, because basic laws of physics dictate that net charge movement across a membrane must always be zero in the steady state, the net ion flux through any one transporter is necessarily joined to all other transporters that carry net charge and affect—and are affected by—voltage across the same membrane. These connections are highlighted in Box 1, which describes diagrammatically the typical H^+^ and charge circuits of the plant plasma membrane. Only by introducing the circuit of a voltage clamp is this interconnection between transporters bypassed so that the individual characteristics of each transport process can be isolated through experimental manipulations such as those used to identify the *Neurospora* H^+^–K^+^ symport. In short, without the voltage clamp, manipulations that alter charge flux through any one transporter necessarily impact on all other charge-carrying transporters in the same membrane, often with unanticipated consequences.

BOX 1. Typical charge and ionic circuits of plant membranes

Illustrated here are the charge and H^+^ circuits of the plasma membrane. A similar set of circuits apply across the tonoplast that incorporate the vacuolar H^+^-ATPase and H^+^-PPase. Physical laws dictate that the net charge flux (grey arrows) across the membrane in the steady state must sum to zero. In other words, at the free-running membrane voltage the net flux of charge out of the cell, here shown primarily as charge movement out through the H^+^-ATPase, must be the same as the net flux of charge back into the cell, here shown as charge movement in through transporters that couple H^+^ return across the membrane with positively- (M^+^) and negatively-charged (X^-^) ions, with uncharged solutes (S) and with respiratory burst oxidases (e^-^) The remaining charge flux is balanced through K^+^, Cl^-^, and Ca^2+^ channels. Note that transport of positively-charged solutes (M^+^) in exchange with H^+^ does not result in net charge movement across the membrane. Such transporters, characterized by cation exchange antiporters of the plasma membrane and tonoplast (Quintero et al., 2000; Pardo et al., 2006; Bassil and Blumwald, 2014), do not contribute to the charge circuit of the membrane. By contrast with the charge circuit, the chemical circuit of the H^+^ (red arrows) need not be balanced across the membrane. Plant cells generally maintain a near-constant pH in the cytosol by H^+^ extrusion through the H^+^-ATPase and H^+^ return through H^+^-coupled transport as indicated, with a major contribution from metabolism (not shown). ATP- and H^+^-coupled transporters are indicated by the green circles, ion channels by the parallel green bars. Solute movements in every case are indicated by the black arrows.

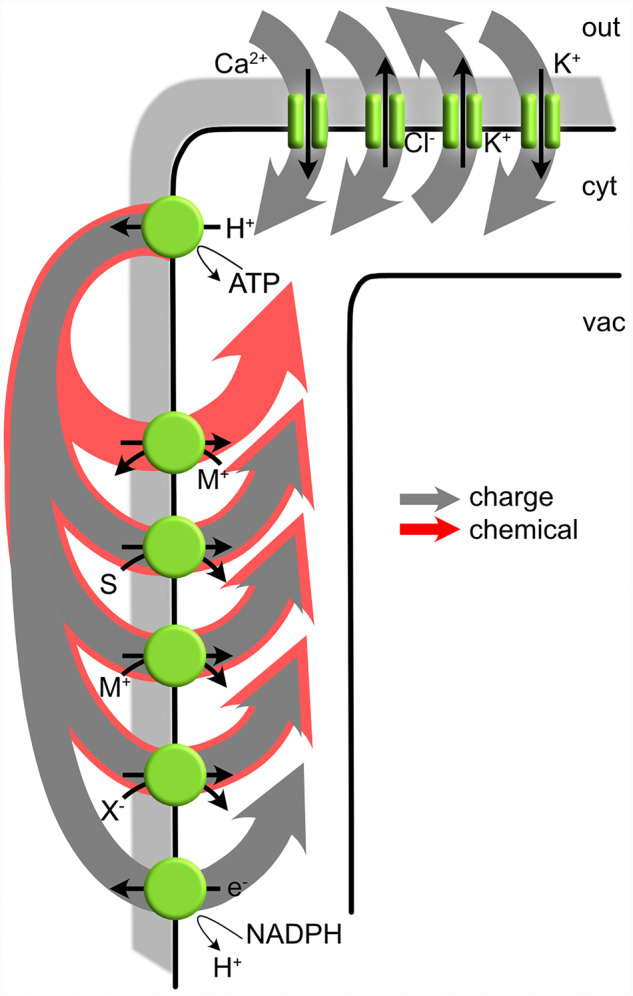

One further example serves to illustrate this point. The SLAC1 Cl^−^ channel was first identified with a mutation in *Arabidopsis thaliana* introducing a high sensitivity to O_3_ and greatly slowed stomatal closure in response to the water-stress hormone abscisic acid (ABA), CO_2_, and dark (Negi et al., 2008; Vahisalu et al., 2008). The *slac1* mutant lacked the slow-activating anion current at the plasma membrane (Schroeder and Keller, 1992), and showed a substantial accumulation of Cl^−^ as well as organic anions in the guard cells. The observations are consistent with the loss of the major pathway for the anion efflux needed to facilitate net osmotic solute and turgor loss from the guard cells, and hence for stomatal closure. However, *slac1* guard cells also accumulated K^+^ in the steady state. Furthermore, they showed a surprising 80% reduction in inward K^+^ current under voltage clamp, and *slac1* stomata also opened roughly three- to five-fold more slowly than in wild-type plants (Wang et al., 2012).

Why should eliminating a Cl^−^ channel, ostensibly associated with stomatal closure, affect K^+^ uptake and stomatal opening? The answer lies in the connection to membrane voltage and, hence, to [Ca^2+^]_i_ and cytosolic pH. Although SLAC1 activity is much reduced except during stomatal closure, it still contributes a background conductance to the membrane. Wang et al. (2012) showed that eliminating this conductance led to a hyperpolarization of the membrane sufficient to promote Ca^2+^ influx through the hyperpolarization-activated Ca^2+^ channels (Grabov and Blatt, 1998; Hamilton et al., 2000) and elevate [Ca^2+^]_i_ substantially above normal resting levels by promoting endomembrane Ca^2+^ release. In turn, the elevated [Ca^2+^]_i_ led to inactivation of the K^+^ channels, which are normally sensitive to [Ca^2+^]_i_ (Grabov and Blatt, 1997, 1999). Similarly, as Cl^−^ and malate accumulated in the guard cell, they suppressed H^+^-coupled Cl^−^ uptake and malic acid synthesis, thereby reducing the H^+^ load on the cell and raising the cytosolic pH (Wang et al., 2012). Again, the K^+^ channels are sensitive to cytosolic pH and their activity declines with the H^+^ concentration (Blatt, 1992; Grabov and Blatt, 1997; Suhita et al., 2004).

There are several insights to be gained from this study. The first and most obvious is of voltage as a shared intermediate, in this case, shared between SLAC1 and the Ca^2+^ channels that trigger [Ca^2+^]_i_ elevations. As a corollary, the connections that arise through [Ca^2+^]_i_ and changes in the cytosolic H^+^ load have far-reaching consequences for metabolism as well as membrane transport, much of which is subject to both cytosolic pH and [Ca^2+^]_i_. Additionally, an all-important message here is that transport across one membrane can affect transport at a second membrane via changes the contents of the shared (cytosolic) compartment (Horaruang et al., 2020). It is worth noting, too, that the *slac1* mutant does not affect stomatal movements in response to changes in atmospheric relative humidity, and this difference in behavior is a predictable consequence of differences behind the mechanics of guard cell turgor (Wang et al., 2017; Pantin and Blatt, 2018), whereas the osmotic water flux for stomatal closure in the dark and on ABA treatments is dominated by ion transport, changes in atmospheric humidity affect the water potential outside the guard cells directly, in the first instance without a substantial transmembrane flux of ions and, thus, separate from alterations in ion channel activities per se.

## Voltage contributes to signal transmission through its action on transport

The fundamental nature of voltage as a shared intermediate among transporters in a membrane is equally relevant to its roles in signal transmission and transduction. Because voltage changes invariably alter the transmembrane flux of ions through several transport processes, they are bound to affect the way in which these changes are transmitted, both in time and space. In other words, the consequence for a cell of a voltage change depends on *how* voltage alters ion flux and, in turn, its impact on the ion content of the cell. There is nothing mysterious here. Nor do such electrical characteristics imply any higher sensory function, as Alpi et al (2007) and Taiz et al (2019) have noted. The role of voltage in signaling is almost always simply one of transmitting and transducing information by modifying the ionic characteristics within cells and tissues.

The propagation of neuronal APs (Jack et al., 1983; Hille, 2001) is a classic example of signal transmission and its transduction to engage cellular processes. In nerve, local depolarizations—so-called excitatory post-synaptic potentials—shift the membrane into the voltage range that activates Na^+^ channels to allow for Na^+^ influx, thereby promoting and propagating the depolarizing voltage change along the length of the neuron in the form of an AP. The Na^+^ flux with each AP is very small and has little effect on the Na^+^ content of the neuron. Likewise, the depolarized voltage during the AP activates voltage-gated K^+^ channels that mediate a K^+^ efflux to repolarize the membrane, and again the flux of K^+^ during the recovery phase of each AP is very small. Together, these alkali cation fluxes are soon compensated by Na^+^ extrusion and K^+^ uptake via the Na^+^/K^+^-ATPase. However, when the voltage depolarization reaches the nerve terminal, it activates voltage-gated Ca^+^ channels that are localized at the presynaptic membrane, enabling a Ca^2+^ influx that greatly elevates [Ca^2+^]_i_. It is the rise in [Ca^2+^]_i_ that transduces the voltage signal by promoting Ca^2+^ binding to synaptotagmins at the inner surface of the presynaptic membrane thereby facilitating vesicle fusion with membrane and neurotransmitter release.

There are a few noteworthy exceptions to the voltage-ion flux connection in which voltage affects cytosolic biochemistry directly. These include a small group of voltage-coupled phosphatases in ascidian eggs that are anchored to the membrane via a protein domain with high homology to the voltage sensor domains of many ion channels (Murata et al., 2005; Iwasaki et al., 2008). Additionally, the production of reactive oxygen species (ROS) itself may be subject to membrane voltage changes. Respiratory burst (NADPH) oxidases of plants, like those of animals, transport charge across membranes (Kimball and Saier, 2002; Luethje et al., 2013; Picco et al., 2015). At the plasma membrane, therefore, such activities will be subject to voltage (Box 1) and voltage changes may impact directly on ROS release. Whether oxidase activity alters membrane voltage is not clear, however, and what evidence exists for voltage changes driven directly by oxidases in plants (Marhavy et al., 2019) is questionable in the absence of voltage clamp measurements (DeCoursey et al., 2000; Reeves et al., 2002). Best estimates of the rates of NADPH oxidation—around 5–10 nmol min^−1^ mg protein^−1^ (Demaurex and Petheo, 2005; Zhang et al., 2018)—translate to a transmembrane current of less than 2% of the total current circulating across the plasma membrane in a typical mesophyll cell of 1.7 ng protein cell^−1^ (Heinemann et al., 2020). In short, the conductances introduced by activating respiratory burst oxidases are likely to be too small to have a substantial effect on membrane voltage.

## Classifying voltage transients

Stimuli both external and internal to a cell can, and very often will alter the flux of one or more ions, thereby affecting the balance of all ion flux which, in turn, will alter the transmembrane voltage. Changes in voltage may arise with altered transport across an endomembrane—for example as a consequence of mutations that affect endomembrane Ca^2+^ release from endoplasmic reticulum or vacuolar stores (Peiter et al., 2005; Vincent et al., 2017)—but because such changes generally affect the ionic contents of the cytosol, they will also affect ion flux at the plasma membrane and, hence, the plasma membrane voltage. Indeed, virtually all phenomenological descriptions of voltage changes, or transients, in plants relate to the plasma membrane, whether or not their origins are to be found first in an ion flux at the plasma membrane or at an endomembrane. Similarly, all forms of electrical activity, whether confined to a single cell or transmitted through a tissue, can be traced back to ion flux changes within a single cell.

Voltage transients in plants are frequently divided between a variety of electrical activities, distinguished primarily by their temporal characteristics and spread through the tissue. Such phenomenological definitions are not always informative and can lead to misconceptions, for example, that membrane voltage is an entity in its own right, disconnected from ion transport. We favor definitions based on the underlying mechanics of transmission, thereby keeping sight of the biology that generates this activity and its consequences. With the exception of APs, however, the mechanics of voltage transients in plants remain poorly defined (Table 1).

**Table 1:** Characteristics of voltage transients and their mechanics in plants

Characteristic	AP	VP	SP
Voltage threshold	Yes	No	No
Spatial propagation	Indefinite	Decays	Indefinite
Propagation rate	0.5–4 cm s^−1^	<0.1 cm s^−1^	≪0.1 cm s^−1^
Typical duration	3–20 s	≫60 s	≫60 s
Mechanism	ion channel activation	uncertain	H^+^-ATPase activation^a^
Voltage direction	Depolarization	Depolarization	Hyperpolarization
Typical amplitude	120–150 mV	3–30 mV	20–50 mV
Dominant ions	Cl^−^ and K^+^ efflux	Uncertain^a^	Uncertain^a^
Modulation	No	Yes^a^	Yes^a^

a May incorporate changes in background membrane conductance for several ions.

*Action Potentials* (APs) are fast “all-or-nothing” voltage transients and the only transients that are described quantitatively with a clearly defined mechanism. Key characteristics of APs are their initiation on passing a threshold in local voltage, their temporal progression through depolarizing and recovery phases, and their rapid self-propagation across long (indeterminate) distances without a loss in peak amplitude. These characteristics arise from positive feedback that is intrinsic to the ion flux carrying the depolarizing phase of the AP—a so-called negative slope to the ion conductance and its time-dependent inactivation—that gives rise to a domino-like propagation of the ion flux along the length of a cell (Jack et al., 1983). In all plant cells examined to date, whether giant algae (Findlay, 1959; Beilby and Coster, 1979; Thiel et al., 1993; Homann and Thiel, 1994; Thiel and Dityatev, 1998; Wacke and Thiel, 2001), root tissues (Felle and Zimmermann, 2007; Furch et al., 2009), or guard cells (Gradmann et al., 1993; Blatt and Thiel, 1994; Grabov and Blatt, 1998; Chen et al., 2012; Minguet-Parramona et al., 2016), the depolarizing phase of the AP is triggered by a Ca^2+^ influx and rise in [Ca^2+^]_i_ that activates SLAC1-like Cl^−^ channels, and especially the strongly voltage-gated ALMT-type anion channels (Meyer et al., 2010; Eisenach et al., 2017; Wang et al., 2017) at the plasma membrane as well as suppressing the activity of the H^+^-ATPase (Smith and Beilby, 1983; Chen et al., 2012; Minguet-Parramona et al., 2016). Both the Cl^−^ efflux and the reduced H^+^ efflux thus affect charge flux for membrane depolarization. In turn, membrane depolarization reduces [Ca^2+^]_i_ by suppressing Ca^2+^ influx and promoting Ca^2+^ resequestration, thereby removing the stimulus for the Cl^−^ flux; it also activates outward-rectifying K^+^ channels for K^+^ efflux, which repolarizes the membrane. Whereas APs of nerve occur over timescales of milliseconds, plant APs progress through triggering, depolarization, and repolarization over seconds or longer. Unlike the nerve AP, plant APs carry a wave of elevated [Ca^2+^]_i_ and do not depend on time-dependent inactivation of the main, charge-carrying Cl^−^ and K^+^ conductances. Also by comparison with APs in animals, which are osmotically neutral, plant APs result in a net loss of osmotic solute and are clearly important for osmotic adjustment in the Characeae (Thiel et al., 1997; Shepherd et al., 1999; Beilby and Shepherd, 2001) and in guard cells to drive stomatal closure (Chen et al., 2012; Minguet-Parramona et al., 2016).

*Variation potentials* (VPs) are generally described as slow voltage waves, often evoked by local tissue damage such as wounding (Felle and Zimmermann, 2007; Zimmermann et al., 2009). Like APs, VPs are depolarizing voltage transients and, in some circumstances, may trigger APs. However, VPs often last for many minutes; they are not recurring nor have a defined temporal characteristic; and their amplitude depends on the stimulus. The mechanisms generating VPs are not well-defined and may occur within a cell either, or both, as the result of H^+^-ATPase inactivation and of Cl^−^ or K^+^ channel activation, and are likely associated with changes in other ion fluxes, including of Ca^2+^. One probable cause of VPs is an initial, local change in the apoplast solute composition, such as may result from tissue damage and leakage of solutes, and on programmed cell death (Garcia-Mata and Lamattina, 2001; Howe and Jander, 2008; Boursiac et al., 2010; Hilleary and Gilroy, 2018). VPs are not self-propagating. Although they may be transmitted over distances of a few cell diameters via plasmodesmatal connections (Spanswick, 1972; Overall and Gunning, 1982), the voltage transient—and its impact on transport (see Voltage is a shared kinetic intermediate in transport)—decays with distance from the point of stimulus.

*System potentials* (SPs), like VPs, are slow voltage waves of variable amplitude, but unlike VPs are characterized by membrane hyperpolarizations that may propagate long distances through the plant (Zimmermann et al., 2009). Based on their sensitivity to H^+^-ATPase antagonists, SPs have been proposed to be mediated by local H^+^-ATPase activation. Other mechanisms that have yet to be explored from the standpoint of voltage include hormone- and environmentally evoked vesicle traffic to and from the plasma membrane, including that of H^+^-ATPases (Hager et al., 1991; Xia et al., 2019, 2020) and ion channels (Sutter et al., 2006, 2007), that affect the population of transporters at the membrane. SPs may be triggered also by local changes in the apoplast concentrations of these ions, thereby altering the conductances to the ionic species in addition to H^+^. However, like any change in transport activity across a common membrane (see Voltage is a shared kinetic intermediate in transport), the consequence is to alter the balance of flux of other solutes, including that of Cl^−^, K^+^, and Ca^2+^. Such interactions confound the distinction between cause and effect unless the individual transport activities are dissected using quantitative voltage-clamp methods. Similarly, the mechanism of SP propagation is not understood, but these transients are sufficiently slow that they may depend locally on chemical propagation (Monshausen et al., 2009; Gilroy et al., 2016).

## Propagation and physiological integration of voltage signals

A very long list of studies link physiological and developmental responses in plants to changes in voltage, often in conjunction with [Ca^2+^]_i_ as well as cytosolic pH and ROS (Tables 2 and 3), even if few have been resolved with any quantitative detail from stimulus, through voltage transients to the response. A selection of examples bears mention here.

**Table 2:** Voltage transients and their functions in plants

Transient	Stimulus	Allied physiology	Selected citations
AP	Temperature	Nyctinastic movements, phloem transport, photosynthesis, osmotic adjustment	^a^
	Electro/mechanical	Gas exchange, osmotic adjustment, cell expansion, nyctinastic movements	^b^
	Drought, salt, hormones	Gas exchange, osmotic adjustment, stomatal movements, photosynthesis	^c^
	Herbivory, wounding	Defense responses, gene expression	^d^
VP	Temperature	Nyctinastic movements, osmotic adjustment, photosynthesis	^e^
	Wounding	Nyctinastic movements, defense responses, photosynthesis, gene expression	^f^
	Drought, salt	Defense responses, gene expression	^g^
SP	Temperature, salt, drought, organics, wounding	Defense responses, gene expression	^h^

a Beilby and Coster (1976); Sanders (1981); Fromm and Bauer (1994); Fromm and Lautner (2007); Grams et al. (2009); Fromm et al. (2013).

b Sibaoka (1969); Williams and Pickard (1972); Abe and Oda (1976); Edwards and Pickard (1987); Sibaoka (1991); Thiel et al. (1993); Grabov and Blatt (1998); Stankovic et al. (1998); Grabov and Blatt (1999); Shimmen (2001); Favre and Agosti (2007); Volkov et al. (2008); Minguet-Parramona et al. (2016).

c Findlay (1959); Williams and Pickard (1972, 1972); Abe and Oda (1976); Blatt and Armstrong (1993); Gradmann et al. (1993); Blatt and Thiel (1994); Fromm and Bauer (1994); Homann and Thiel (1994); Grabov and Blatt (1998); Thiel and Dityatev (1998); Grabov and Blatt (1999); Garcia-Mata et al. (2003); Wang et al. (2013).

d Stankovic and Davies (1996); Favre et al. (2001); Whalley et al. (2011); Fromm et al. (2013); Mousavi et al. (2013); Whalley and Knight (2013); Zimmermann et al. (2016).

e Filek and Koscielniak (1997); Koziolek et al. (2004); Lautner et al. (2005); Galle et al. (2013); Lautner et al. (2014); Vuralhan-Eckert et al. (2018).

f Stankovic and Davies (1996); Herde et al. (1999); Vodeneev et al. (2012); Fromm et al. (2013); Galle et al. (2013); Salvador-Recatala et al. (2014); Nguyen et al. (2018); Toyota et al. (2018).

g Favre et al. (2001); Mousavi et al. (2013); Zimmermann et al. (2016).

h Zimmermann et al. (2009, 2016).

**Table 3:** Voltage-associated ionic and chemical second messengers

Second Messenger	Stimulus	Response	Selected citations
H^+^	Light, mechanical, salt/osmotic stress, hormone, nutrient	Altered growth, stomatal movement, root development	^a^
	Fungal interaction, wounding	Pathogen defense, altered growth	^b^
Ca^2+^	Drought, salt, osmotic, stress, mechanical, thermal stress	Gene expression cyclosis, nyctinasty, dormancy, thermal adaptation stomatal movement, gene expression	^c^
	Pathogen/elicitor interaction, wounding	Pathogen defense, altered growth	^d^
Ca^2+^, H^+^	Electrical, hormone	Stomatal movement, root gravitropism, root hair/pollen growth gene expression	^e^
Ca^2+^, ROS, Nitric Oxide	Drought, salt, osmotic thermal stress, pathogen/elicitor interaction	Altered growth, gene expression, pathogen defense	^f^
	Drought, salt, osmotic stress, hormone	Stomatal movement	^g^

a Blatt (1992); Blatt and Armstrong (1993); Hoth et al. (1997); Amtmann et al. (1999); Geilfus et al. (2015); Fendrych et al. (2016).

b Felle and Zimmermann (2007); Felle et al. (2009).

c Lynch et al. (1989); Knight et al. (1997); Grabov and Blatt (1998, 1999); Hamilton et al. (2000); Kiegle et al. (2000); Hamilton et al. (2001); Chen et al. (2010); Whalley et al. (2011); Whalley and Knight (2013); Choi et al. (2014); Liu et al. (2020).

d Blume et al. (2000); Zimmermann et al. (2009); Thor and Peiter (2014); Nguyen et al. (2018); Toyota et al. (2018).

e Blatt and Armstrong (1993); Thiel et al. (1993); Herde et al. (1995); Stankovic and Davies (1996); Grabov and Blatt (1997); Stankovic and Davies (1997); Herde et al. (1999); Monshausen et al. (2007, 2009, 2011); Michard et al. (2011).

f Durner et al. (1998); Kovtun et al. (2000); Lecourieux et al. (2002); Lamotte et al. (2004); Evans et al. (2016); Choi et al. (2017); Hilleary and Gilroy (2018); Toyota et al. (2018).

g Pei et al. (2000); Neill et al. (2002); Garcia-Mata et al. (2003); Kwak et al. (2003); Sokolovski and Blatt (2004); Bright et al. (2006).

*Leaf movements* of the sensitive plant *Mimosa pudica* and the Venus flytrap, *Dionea muscipula*, have long been known to arise in association with electrical signal transmission (Sibaoka, 1969). Similar nyctinastic leaf movements are known in other species as well, including *Samanea saman* and the bean *Phaseolus vulgaris*. A feature of these leaves is their specialized anatomical arrangements of cells within a “hinge” region, or pulvinus, at the base of the leaf. A similar mechanical arrangement is present in the Venus flytrap, in which the bistable structure at the base of the trap deforms and leads to a rapid, “snapping” closure of the trap (Forterre et al., 2005; Morris and Blyth, 2019; Sachse et al., 2020). In *Mimosa* and the Venus flytrap, mechanical stimulation triggers the generation of APs that propagate through the tissues to trigger solute and water fluxes from the pulvinar tissues (Simons, 1981) and these fluxes in turn allow for the mechanical displacements of the tissue. The studies of Ruth Satter, Arthur Galston, Youngsook Lee, and Nava Moran through the 1970s and 1980s (Satter and Galston, 1971, 1981; Satter et al., 1974; Lee and Satter, 1987; Moran et al., 1988; Moran and Satter, 1989) identified several of the ion channels and H^+^-ATPase characteristics, establishing their contributions to the slower pulvinar ion fluxes and their regulation in *Samanea*. Studies of the mechanosensitive *Mimosa* pointed to similar contributions of K^+^ and Cl^−^ fluxes in these leaf movements (Abe, 1981; Stoeckel and Takeda, 1993).

The rapid and large mechanical displacements of the Venus flytrap pose greater technical challenges but are clearly associated with very similar ion and water fluxes (Volkov et al., 2008). Early work demonstrated that mechanical stimulation of specialized trigger hairs give rise to APs that propagate across the leaf (Stuhlman and Darden, 1950) and that a threshold of stimulation, often requiring two or three displacements of a trigger-hair, was needed to close the trap (Dipalma et al., 1961). These observations have since been lauded as evidence of “counting” by the plant, although there is nothing unique to this phenomenology. Additive inputs that sum to exceed the threshold for excitation are common to the triggering of APs generally [cf. Jack et al. (1983), Burri et al. (2020), and Shimmen (2001)]. Furthermore, Venus flytrap APs can be triggered by wounding (Pavlovic et al., 2017), indicating that neither trigger hair displacements per se, nor their repeated disturbance, are prerequisite for stimulation. The nature of the Venus flytrap AP, like that of other leaf movements, remains less well defined. Most likely, these events arise from transient fluxes of Ca^2+^ that elicit much larger fluxes of Cl^−^ and K^+^ (Sibaoka, 1991), as occur in several model plant cell systems, especially in guard cells (below) and *Chara* (above).

Intriguingly, the Venus flytrap utilizes chemicals present in the trap to stimulate secretions over a period of many days that ultimately lead to digestion of the prey (Jaksova et al., 2020). Secreted protein analysis, and recent comparative genomics, indicate an association with pathogenesis- and defense-related proteins, suggesting a common origin of insectivory with defense-related processes (Schulze et al., 2012; Palfalvi et al., 2020). Whether electrical excitation contributes to the secretory process, and if so then how, remain open questions.

*Guard cell* [Ca^2+^]_i_ increases are commonly associated with stimuli leading to stomatal closure (Roelfsema and Hedrich, 2010; Jezek and Blatt, 2017). The mechanistic connection to membrane voltage was made by Grabov and Blatt (1998), who reported cyclic [Ca^2+^]_i_ increases in guard cells, recorded with the Ca^2+^-sensitive dye Fura2, when the plasma membrane hyperpolarized and quantified this dependence under voltage clamp. Their studies led to the identification of the dominant, voltage-activated Ca^2+^ channel in the guard cell plasma membrane that is activated by the water-stress hormone ABA (Hamilton et al., 2000, 2001) and by redox stress (Pei et al., 2000), and to its role in triggering endomembrane Ca^2+^ release (Grabov and Blatt, 1999; Garcia-Mata et al., 2003). Grabov and Blatt (1997, 1999) defined the kinetics of the inward-rectifying K^+^ channels and their dependence on [Ca^2+^]_i_, and later work (Mori et al., 2006) led to similar quantifications of guard cell anion channels with [Ca^2+^]_i_ (Chen et al., 2010; Meyer et al., 2010; Stange et al., 2010).

Guard cells exhibit cardiac-like APs (Blatt and Armstrong, 1993; Gradmann et al., 1993; Blatt and Thiel, 1994). Given that membrane depolarizations are associated with [Ca^2+^]_i_ elevations (Grabov and Blatt, 1998), it followed that these voltage oscillations should be coupled with [Ca^2+^]_i_ and with a cyclic release of K^+^ and Cl^-^ (Blatt, 2000). A voltage-[Ca^2+^]_i_-osmotic flux cycle is one of the many predictions, since validated experimentally, arising from the OnGuard systems platform that incorporates the wealth of data for guard cell transport and cellular homeostasis (Chen et al., 2012; Hills et al., 2012; Wang et al., 2017). This flux cycle leads to oscillatory “bursts” in solute efflux when the membrane depolarizes and K^+^ and Cl^−^ are lost, thereby promoting stomatal closing (Minguet-Parramona et al., 2016). Indeed, the step-wise closure of stomata has been observed, associated with Ca^2+^ availability outside (Yang et al., 2006) and, hence, most likely with its ability to trigger intracellular Ca^2+^ release. The temporal characteristics of voltage changes in the guard cell are a critical factor also determining the success of the optogenetic K^+^ channel, BLINK1, to accelerate stomatal opening and closing, thereby enhancing carbon assimilation while also increasing whole-plant water use efficiency (Papanatsiou et al., 2019).

One central conclusion to come from analyzing these experiments is that voltage and [Ca^2+^]_i_ oscillations are tightly interconnected with the kinetics of K^+^ and Cl^−^ flux. The temporal characteristics of the oscillations in voltage and [Ca^2+^]_i_ during stomatal closure thus “reflect a spectrum of frequencies that emerge from the balance of intrinsic transport activities” in the guard cell (Minguet-Parramona et al., 2016). In short, these studies are an excellent example of membrane voltage as a platform that integrates each of the fluxes, Ca^2+^, K^+^, and Cl^−^, for osmotic regulation. They provide the only example in plants for which this integration is fully detailed with quantitative kinetics.

*Systemic stomatal signaling* is also evident in response to environmental stimuli, including light and the partial pressure of CO_2_ (pCO_2_). Very high fluence rates incident on one leaf, for example, will trigger stomatal closure and can lead to a wave of ROS that promotes closure of stomata on other leaves of the same plant (Devireddy et al., 2018, 2020). Similar long-distance responses are also evident with step changes in pCO_2_ (Ehonen et al., 2020). In Arabidopsis, the ROS signal travels at speeds around 0.05–0.1 mm s^−1^ and depends on expression of the respiratory burst oxygenase gene *RBOHD* with impacts on cellular metabolism, including glucose, sucrose, and Krebs cycle intermediates (Choudhury et al., 2018). Here, the spatial transmission of voltage changes may also impact directly on ROS release, plausibly through a voltage-dependence in electron transport by the respiratory burst oxidase (see Voltage contributes to signal transmission through its action on transport above).

Not surprisingly, systemic stomatal signaling associates also with the SLAC1 Cl^−^ channel and the OST1 protein kinase (Devireddy et al., 2018). The relevance of these gene products to transmission, rather than the final stomatal response, is not clear. While these studies do not address the physiological mechanism of ROS wave propagation, the speed of transmission and its parallel to similar phenomena involving long-distance signaling, including herbivory (below) and pathogen infection (Gilroy et al., 2016; Melotto et al., 2017), suggest mechanisms that include VPs, possibly in combination with APs and their associated [Ca^2+^]_i_ transients, and may depend in part on transmission through the vascular tissue between leaves.

*Herbivory* by insects has long been known to trigger systemic defense responses in plants (Howe and Jander, 2008), including alterations in gene expression in distant parts of the plant. Such systemic responses have been associated with SP transmission (Felle and Zimmermann, 2007; Zimmermann et al., 2009), ROS (Gilroy et al., 2014; Hilleary and Gilroy, 2018), and more recently, with glutamate receptor-like (GLR) channels. Toyota et al. (2018) and Nguyen et al. (2018) demonstrated that long-distance propagation of a systemic signal, whether herbivory, wounding, or mechanical disturbance, is accompanied by a wave of elevated [Ca^2+^]_i_ through the surrounding tissue that can be resolved with the fluorescent Ca^2+^ sensor GCaMP3. The [Ca^2+^]_i_ wave usually propagates at roughly the speed of an electrical signal (the relative contributions from AP, VP, and/or SP propagation is not clear) from the site of stimulus, through the petiole, and into leaves most closely connected through the vasculature.

Toyota et al. (2018) proposed that transmission of the [Ca^2+^]_i_ wave is mediated, cell-to-cell, through plasmodesmata. Experimental analysis of transmission (Evans et al., 2016) indicated that the [Ca^2+^]_i_ wave depends on chemical propagation through the release of, and reaction to ROS, and modeling suggested that cell-to-cell transmission might be associated with Ca^2+^ diffusion through plasmodesmata. However, the evidence supporting such a mechanism is equivocal, as long-distance signaling was supported by either of two plasma membrane-localized GLR channels, *GLR3.3* and *GLR3.6*, that are differentially localized to the xylem and phloem (see also Nguyen et al., 2018). Only in the *glr3.3glr3.6* double mutant was propagation of the [Ca^2+^]_i_ wave lost although local [Ca^2+^]_i_ elevations were still recorded, indicating the presence of additional pathways for Ca^2+^ flux that are active in these tissues. External applications of glutamate suggested that the amino acid was sufficient to trigger a systemic, GLR3.3/GLR3.6-dependent [Ca^2+^]_i_ wave, the observations implicating a plasma membrane-associated flux of Ca^2+^. However, to further complicate matters, the glutamate used—100 mM—was some two orders of magnitude above the physiological range of concentrations likely in the apoplast, thus raising questions about specificity and its mechanism of action.

Wound signal transmission raises other questions as well. Among these, Evans et al. (2016) have argued in favor of a role for the functionally elusive TPC1 channel. They modeled cell-to-cell transmission incorporating a key role for endomembrane (vacuolar) transport and Ca^2+^ release via TPC1 with secondary consequences for transport at the plasma membrane (see Voltage is a shared kinetic intermediate in transport). One difficulty with this mode of transmission, and the arguments for the TPC1 channel, is that TPC1 localizes to the tonoplast membrane. As mature xylem comprises dead and cleared cell files and mature phloem cells lack the tonoplast, the arguments around the function of this channel will need revisiting. Testing models of cytoplasmic and plasmodesmatal transmission may prove difficult, too, as it will be essential to demonstrate these events in the absence of local changes in ion flux at the plasma membrane.

One final point about the methods of electrical recording deserves a mention here. Both the studies of Toyota et al. (2018) and of Nguyen et al. (2018) made use of surface electrodes to record voltage differences between the points of contact on the petiole or leaf surfaces and a reference point at the root. Recordings of this kind will report changes in voltage that may be related to underlying transport activities in the tissue, but they are fundamentally measurements of the apoplast of the tissue and plant, not of membrane voltage. So, they are not an assured measure of transport activity. Furthermore, in application to multicellular plant tissues, they do not allow the transient to be associated with a specific cell type but only to the tissue as a whole. As a consequence, direct temporal and spatial connections with events taking place within a subset of cells within the tissue, including with [Ca^2+^]_i_ waves, is problematic. In short, without the insights accorded by the voltage clamp, the connection between the electrical signal and the [Ca^2+^]_i_ wave is not clear cut. We must ask, then, whether the GLR channels play a primary or secondary role, much as the *slac1* mutant affects stomatal opening (Wang et al., 2012).

## Longer-term consequences of voltage signals

Gene expression control plays an important part in developmental and longer-term stress responses, notably in induced or acquired resistance (Gurr and Rushton, 2005; Howe and Jander, 2008). Such control is frequently cited as a consequence of long-distance electrical signaling (Felle and Zimmermann, 2007; Hilleary and Gilroy, 2018) even if causality generally has been inferred only. In animals, control of gene expression for neuronal development has been shown to be use-dependent, often tied to Ca^2+^ channel activity (Barbado et al., 2009). The seminal study by Flavell et al. (2006) showed that a high frequency of [Ca^2+^]_i_ elevations with synaptic stimulation activated a calcineurin phosphatase, leading to dephosphorylation of the MEF2 transcription factor and MEF2 activation. In turn, MEF2 promoted the transcription of a subset of genes that restricted the development of synaptic connections. This mechanism is now recognized to connect the electrical activity of neurons with their remodeling as well as influencing the populations of specific ionotropic receptors (Yap and Greenberg, 2018; Kamath and Chen, 2019; Hammond-Weinberger et al., 2020).

In multicellular plants, as noted above, the mechanics of long-distance electrical transmission and its relationship with specific ion fluxes remain poorly defined. Nonetheless, parallel transmission of [Ca^2+^]_i_ and ROS transients provide phenomenological evidence that is tantalizing and provides a link to gene expression. Some of the most convincing studies have come from the Knight laboratory and their analysis of gene expression with [Ca^2+^]_i_ oscillations. Whalley and Knight (2013) showed that imposing a large electrical field on seedlings to drive cyclic changes in [Ca^2+^]_i_ using three alternative voltage sequences enhanced differentially the transcription of a range of stress-associated genes when assayed by whole-genome microarrays. These [Ca^2+^]_i_ oscillations were designed to mimic the responses to external stimuli, including cold and drought, that can evoke distinctive [Ca^2+^]_i_ oscillations, or “signatures”, which differ in frequency and amplitude. Promoter-reporter analysis supported these findings to show a tight regulation of selected genes associated with specific [Ca^2+^]_i_ signatures and complement the [Ca^2+^]_i_-dependencies of a subset of promoter elements (Whalley et al., 2011). In short, even if the connections to electrical activity can only be inferred at present, the consequences in elevating [Ca^2+^]_i_ and its impact on gene expression are firmly established.

How are these [Ca^2+^]_i_ signals decoded? Lenzoni et al. (2018) and Liu et al. (2020) used mathematical modeling and experimental analysis of the expression of selected genes responsive to pathogenesis. They showed that, for the [Ca^2+^]_i_-dependent cascade leading to calmodulin-transcription factor binding, expression control of these genes can be understood to arise from three, intersecting pathways. Each of these signaling pathways operates with distinct and highly nonlinear dynamics, which confers both an exceptionally broad range of gains in transcript amplification and a sensitivity to a well-defined temporal sequence of [Ca^2+^]_i_ oscillations. Equally striking, the analysis suggested that the kinetics of [Ca^2+^]_i_ recovery plays an important part in defining gene expression. In other words, the shape of the [Ca^2+^]_i_ transient is just as important as its frequency and amplitude. Obviously, these studies offer just one set of examples. We can anticipate that substantial complexity may be added in other circumstances to expand the breadth of controls (Cheung et al., 2020).

## Concluding remarks

While the impact of APs on osmotic adjustment is clear cut, the roles for VPs and SPs and the information that they are proposed to transmit, with few exceptions, are inferred by association only. For VPs and SPs, key questions still remain about the underlying mechanics and ion fluxes that define the changes in voltage. In these instances, a point of confusion also lies in distinguishing between cause and effect in relation to the associated ion fluxes, their temporal and spatial kinetics. Because membrane voltage is intrinsically the product of a balance of ion flux, it is important to recognize that voltage changes always represent a change in underlying flux through at least two (and often more) transporters. Thus, deciphering the underlying ion fluxes for VPs and SPs will require quantitative voltage-clamp analysis and cannot be deduced from surface potential measurements. Such studies should also help to resolve the roles for these voltage transients in connection with APs especially as may arise from local VP spread within a plant tissue.

Other than the protein conformational changes that gate voltage-sensitive channels (Dreyer and Blatt, 2009; Jegla et al., 2018) and the *Ciona intestinalis* VSP protein phosphatase, which also incorporates a channel-like voltage sensor domain as a membrane anchor (Murata et al., 2005; Iwasaki et al., 2008), there are very few instances in which voltage itself is coupled directly to the biological response without an ionic intermediate. Most often voltage signals connect to the biology of a cell through ion flux, especially through Ca^2+^ flux that drives changes in [Ca^2+^]_i_. Plant APs are widely recognized to incorporate changes in [Ca^2+^]_i_ as a component in their propagation. Cycles of APs thus are capable of generating oscillatory changes in [Ca^2+^]_i_. As inferred from the preceding discussion, VPs and SPs may also influence [Ca^2+^]_i_ and all three voltage transient forms have the potential to affect cytosolic pH and generate ROS. These ionic and chemical intermediates, in turn, clearly have profound effects within the cell, both over physiological timescales, including on ion transport itself, and also on developmental and long-term adaptive processes that rely on modulated gene expression. We anticipate that further connections will surface in the near future, especially in processes occurring over intermediate timescales that rely on protein delivery and recirculation via membrane traffic (Sutter et al., 2007; Eisenach et al., 2012; Xia et al., 2019).

## Funding

This study was funded by the Biotechnology and Biological Sciences Research Council (BBSRC) research grants BB/P011586/1, BB/L019025/1 and BB/N01832X/1 and by a Lord Kelvin and Adam Smith PhD Studentship to FALS-A

*Conflict of interest statement*. None declared.

**Outstanding Questions**

- For Variation and Systemic Potentials, a quantitative analysis of the transport mechanics is generally lacking and will be needed to fully understand the origins, propagation, and consequences of these transients at the cell and tissue levels. Are these voltage changes associated with a common set of transporters or are their underlying mechanics variable between tissues and species?
- Variation potentials - sometimes called Slow Wave Potentials - may be important to drive the membrane voltage beyond the threshold triggering action potentials, but their impact in signalling requires quantitative analysis, drawing on voltage clamp methods. Is there a generalizable model thay may help define the temporal and spatial relationships between these voltage transients?
- It remains unclear whether transmission of voltage signals between cells is mediated by transport across the tonoplast with cell-to-cell transmission via plasmodesmata, independent of transport across the plasma membrane. Separate analysis of changes in transport activities at both membranes will be vital to decipher these relationships. For example, can the putative roles for reactive oxygen species (ROS) be resolved with sufficient temporal accuracy to separate the actions at the two membranes?
- Transmission of voltage transients over long distances has been associated with ROS, possibly as a mediator in transmitting the voltage signal across cell boundaries. To establish the mechanics of this transmission it will be essential to identify the immediate targets of ROS. What are the quantitative relationships between the generation of ROS and membrane voltage? Can these be resolved through combined voltage-clamp and ROS imaging studies?
- The role of the slow-vacuolar cation channel TPC1 in voltage signal transmission continues to puzzle more than 30 years after the channel was first described. Clarity is needed, with physiological analysis of channel activity that extends beyond mutant analysis, to demonstrate the function of this current. Are there as yet unidentified factors that could explain the lack of TPC1 activity over the known range of physiological voltages at the tonoplast?
- Oscillations in [Ca^2+^]_i_ can encode for selective gene expression. Can similar oscillations in cytosolic pH also play a role and, if so, in what capacity?

## Footnotes

* The distinction here between voltage as a substrate and as a product is somewhat arbitrary. For any transporter that moves a net charge across a membrane, the membrane voltage is a driving force that acts on the charge flux (as a 'substrate'), and the charge flux in turn will affect the membrane voltage (as a 'product'). For example, the plasma membrane H^+^-ATPase uses the energy of ATP hydrolysis to pump H^+^ out of the cell, thereby contributing to a voltage that is negative inside relative to outside the cell. At the same time, this voltage also acts as a driver that reduces H^+^ efflux by opposing the movement of the charged species, H^+^, out of the cell. Indeed, the plasma membrane H^+^-ATPases of fungi and of plant cells show a significant dependence on voltage across the normal physiological voltage range (Blatt, 1987; Blatt and Slayman, 1987; Blatt et al., 1990; Chen et al., 2012).

## Notes

M.K., F.A.L.S.-A., M.R.B., and J.C.A. conceived and developed the review topics, and wrote the review; M.R.B., J.C.A., and F.A.LS.-A. assembled the figures; M.K., F.A.L.S.-A., and J.C.A. assembled the tables; M.R.B. edited the final text.

The author responsible for distribution of materials integral to the findings presented in this article in accordance with the policy described in the Instructions for Authors (https://academic.oup.com/plphys/pages/general-instructions) is: Michael R. Blatt (michael.blatt@glasgow.ac.uk).
